# Distinguishing Reality: A Case of Delusional Misidentification Syndrome in a 39-Year-Old Male

**DOI:** 10.7759/cureus.67001

**Published:** 2024-08-16

**Authors:** Grant Gramling, Michelle Wu, Bishoy Kolta, Shirley Alleyne

**Affiliations:** 1 Osteopathic Medicine, Nova Southeastern University Dr. Kiran C. Patel College of Osteopathic Medicine, Clearwater, USA; 2 Psychiatry Residency Program, Lakeland Regional Health, Lakeland, USA; 3 Medical Education, Lakeland Regional Health, Lakeland, USA

**Keywords:** delusion, psychology, psychiatry, atypical anti-psychotics, schizophrenia, post traumatic brain injury, delusional misidentification syndromes, olanzapine, capgras syndrome, capgras

## Abstract

Capgras syndrome (CS) is a type of delusional misidentification syndrome where an individual is under the impression that a person they know has been switched with an identical imposter. One theory for the development of CS is a disturbance among the frontal, limbic, and temporal areas, which creates an alteration in an individual's ability to recognize a person's face and provoke a response emotionally. The primary risk factors for the development of CS include having a neurological disorder and a diagnosis of schizophrenia. We present a case of a 39-year-old male with a past medical history of traumatic brain injury and familial history of schizophrenia who presented to the Emergency Department with paranoia and the belief that his father had been switched with an imposter. After ruling out organic causes, he was stabilized on olanzapine before discharge to outpatient follow-up. This case highlights the importance of prompt recognition of the symptomatology associated with CS and treatment with olanzapine for a favorable outcome.

## Introduction

Capgras syndrome (CS) is a rare delusional disorder where the affected individual believes that a person they know intimately has been switched with an identical imposter [1]. CS falls into a class of delusional disorders known as delusional misidentification syndromes [1,2]. There are multiple types of this syndrome other than CS; examples include Fregoli delusion, intermetamorphosis, and subjective doubles [2]. Fregoli delusion is a belief where the affected individual believes that strangers or unknown places take on the form of familiar people or places [2,3]. Next is intermetamorphosis, where the affected person believes that someone they know can change either their outward appearance, internal characteristics, or both at will [2]. Lastly, subjective doubles is a belief in which a person thinks there is a duplicate of themselves living somewhere else in the world [2,4].

While the etiology is unknown, CS is commonly associated with brain damage, primarily in the right hemisphere, including the frontal, limbic, and temporal areas [5]. Three psychiatric conditions most commonly associated with CS are schizoaffective disorder, schizophrenia, and bipolar disorder [1]. One underlying theory that explains the manifestation of CS is a disruption between the temporal lobe and the limbic system; this disruption could cause a disconnect between the affected individual’s capacity to recognize faces and their ability to have an emotional response [5]. Additionally, in a recent literature review of CS, not only were schizophrenia, schizoaffective disorder, and bipolar disorder associated with cases of CS, but neurological disorders, such as traumatic brain injuries (TBIs), were also associated with CS [6]. This case report provides further support for neuropsychiatric associations with CS. 

Currently, there are limited evidence-based treatment options for CS. As the literature stands, antipsychotics have shown effectiveness; however, widespread comparisons between different medications for CS have not been thoroughly evaluated [6]. This case report provides an example of an effective treatment, olanzapine, with a positive outcome for this disabling disorder. We present an interesting case of an individual suffering from CS, and we aim to provide more information regarding potential treatments and therapies for this debilitating disease.

## Case presentation

This is a case of a 39-year-old male with no significant past psychiatric history, who presented to the Emergency Department for paranoia and aggressive behavior towards his family in November 2023. Family history was significant for a sister with schizophrenia. He had a past medical history significant for a TBI in 2021, during which he suffered a collision with a train going approximately 40 miles per hour while in his vehicle. On initial arrival following this collision, the patient was unresponsive, with a Glasgow Coma Score of 3. His injuries included intraparenchymal hemorrhage, subarachnoid hemorrhage, secondary adrenal insufficiency, and multiple organ damage, requiring hospitalization in the trauma intensive care unit for 28 days.

**Figure 1:**
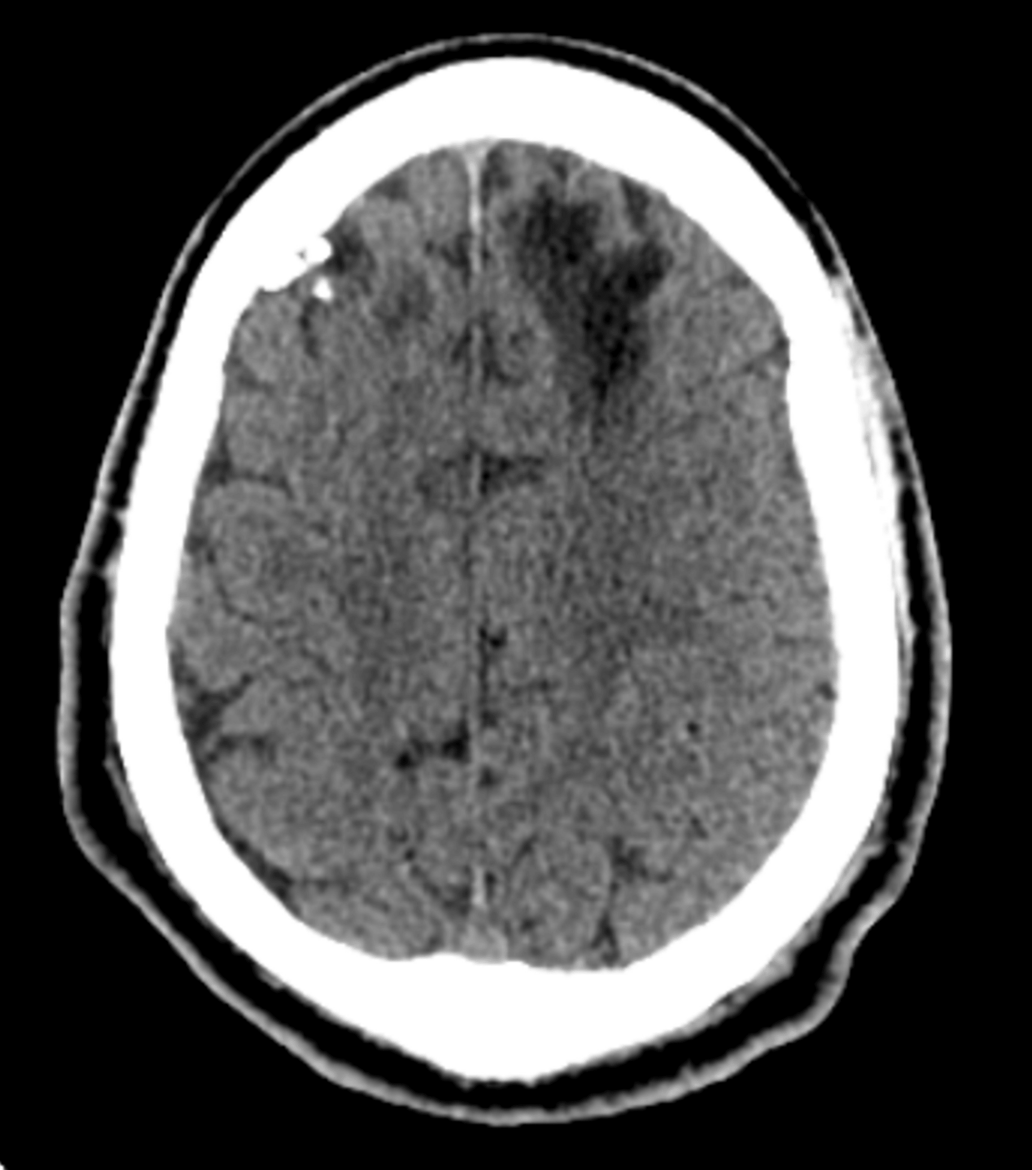
CT of the head without contrast showing the chronic hypodense focus of encephalomalacia in the left frontal lobe. Normal size and configuration of ventricles and cisterns; no mass effect or midline shift.

His mother brought him to the Emergency Department because he had been experiencing paranoia and visual hallucinations for the past six months. His symptoms worsened over the past few weeks, becoming debilitating for his personal and social life; he was unable to hold a job and refused to eat or drink due to his paranoia. During these weeks, he believed that his immediate family, specifically his father, was an imposter. He also presented with episodes of aggression, during which he would break furniture to "kill those imposters", attacked his father with a knife, forced himself into a room, was scared to go outside, and endorsed intermittent homicidal ideation towards his family members. Upon presentation to the Emergency Department, he was alert and oriented but stated that his father had been replaced with a man "wearing a costume and dressed as (his) father", and that although he resembled his father, he was an imposter. Due to his initial presentation, a CT of the head without contrast, chest X-ray, EKG, and a urine drug screen were ordered to rule out organic causes of his progressively worsening symptomatology. The CT without contrast revealed a chronic focus of encephalomalacia in the left frontal lobe, likely from his TBI (Figure 1). However, there was no evidence of any focal neurological deficits. The chest X-ray did not show signs of infection, masses, fluid, or other primary organic causes that could lead to psychosis. His EKG was unremarkable. The urine drug screen was positive for cannabinoids. When asked, the patient admitted to occasional use of tetrahydrocannabinol and denied other drug use. The patient also reported a prior alcohol history of consuming a bottle of Hennessy a day, which he quit a few years ago.

Upon presentation to our Behavioral Health Department, he was seen mumbling to himself and was internally preoccupied. He made statements that he would "kill others to protect himself" and that he was experiencing active auditory hallucinations of "random people" telling him to end his life. His speech was initially disorganized and tangential; however, throughout his stay, he was able to be redirected. Over the first two days of admission, his paranoia and homicidal thoughts about his family worsened in intensity. We initiated a trial of olanzapine for his psychosis and titrated it over three days to 15 mg daily before discharge. After three days of this regimen, his symptoms showed signs of improvement. There were very few mentions of imposters, and his speech had slowly become linear and logical. He was no longer homicidal, and his judgment showed signs of steady improvement. He understood that he would need scheduled outpatient follow-ups to reassess his symptoms, in hopes of complete resolution of symptoms in the coming weeks.

## Discussion

CS presents a unique challenge for management because it is not defined in the Diagnostic and Statistical Manual of Mental Disorders (DSM)-5, and there are no evidence-based guidelines on treating this disorder [1]. Potential reasons why CS and delusional misidentification syndromes are not listed in the DSM-5 could be the lack of empirical data concerning these syndromes, their rarity, and the difficulty in differentiating them from other delusional disorders. There has also not been a proven mainstay treatment for CS. There has been promise regarding the effectiveness of olanzapine and other antipsychotics in past case studies and literature reviews, but olanzapine has not been widely compared to other atypical antipsychotics [6,7]. The effectiveness of olanzapine in the case of our patient adds to the literature related to its positive outcome in CS.

In a similar case study, olanzapine had shown the ability to drastically improve psychotic symptoms, just like in our patient [7]. Olanzapine works as a D2 receptor antagonist in the mesolimbic system, blocking both dopamine and serotonin binding in the post-synaptic cleft [8]. The mesolimbic pathway is an important regulator in transporting dopamine from the ventral tegmental area (VTA) to many areas of the brain, including, but not limited to, the nucleus accumbens, amygdala, and prefrontal cortex [9]. The mesolimbic pathway is also an important regulator of the reward center of the brain and has additionally been shown to play a role in the novelty of a person's personality and impulsivity [9]. This could be one reason why olanzapine should be considered an effective treatment modality for CS. The pathology of the disruption between the temporal lobe and the limbic system and the mechanism of blocking the D2 receptor in the mesolimbic system should be further investigated. In our study, the patient's CT finding of chronic encephalomalacia in the frontal lobe due to a previous TBI exemplifies the associations between CS and neurological disorders. In addition, due to the rarity of CS, more research is needed to identify additional treatment options for those who are unable to tolerate an antipsychotic such as olanzapine.

In a different case study, a patient had failed a trial of olanzapine for ongoing psychiatric issues, as well as other antipsychotics and electroconvulsive therapy, and was given the diagnosis of treatment-resistant schizophrenia [10]. He showed a favorable outcome on clozapine [10]. This supports the treatment of CS with atypical antipsychotics but adds another variable regarding whether olanzapine has been trialed and failed in the past [10].

An additional case study was conducted in a patient with psychosis due to hypothyroidism and myxedema madness, who showed a favorable response to olanzapine as well [11]. This patient’s CS and psychosis were uncontrolled until a trial of olanzapine was initiated [11]. After a combination of olanzapine and levothyroxine, the patient was able to return to her baseline in three weeks [11].

This case report adds to the current, limited literature related to CS because of the positive outcome associated with this patient. While it has been reported in a literature review on CS that 32% of CS cases were linked to schizophrenia and 43% of CS cases were linked to neurological disorders [6], this case demonstrates the importance of properly identifying CS versus other delusional disorders and how prompt treatment can directly contribute to more favorable outcomes and a better quality of life for patients. Once our patient was started on a trial of olanzapine and titrated to 15 mg, he was seen to show adequate clinical improvement. This case was interesting due to its clinical presentation, the aforementioned imaging, and the improvement of symptoms on olanzapine.

Our patient exhibited both a familial history of schizophrenia and a history of a TBI, which supports previous findings of these neuropsychiatric associations in CS. The uniqueness in our case is best underscored by the patient's sister having schizophrenia (not the patient himself), his CT finding of encephalomalacia in the frontal lobe, and the positive outcome due to the commencement of olanzapine. Taking into account the entire patient history is one of the most important factors in managing patients with CS. This includes a detailed familial, past medical, psychiatric, and trauma history for early diagnosis and treatment. Our case further demonstrates that prompt identification of CS and administration of olanzapine can result in a favorable outcome (Table 1).

**Table 1:** Key points and clinical pearls Table credit: Dr. Grant Gramling [1,6]

Key points and clinical pearls
Capgras syndrome is a rare delusional misidentification syndrome that is associated with neurological disorders, schizophrenia, schizoaffective disorder, and bipolar disorder.
Antipsychotics, including olanzapine, are emerging as one of the leading therapeutic medications for the resolution of symptoms of Capgras syndrome.
Taking a detailed patient history is paramount to the correct identification of psychiatric conditions.
Additional research should be conducted in comparing atypical antipsychotic outcomes associated with Capgras syndrome.

## Conclusions

In conclusion, CS should be at the forefront of the clinician’s mind when an individual presents with paranoia and delusions that their loved ones have been switched with an imposter. Knowing the individual’s past medical history, psychiatric history, and family history can be beneficial in aiding diagnosis. In our case report, we found success with olanzapine, as our patient improved significantly and was shortly discharged to outpatient follow-ups. Due to the rarity of this syndrome, the lack of a definition by the DSM-5, and the lack of empiric guidelines for treatment, more research should be conducted into the syndrome as a whole. Additionally, it would be beneficial to add more comparisons of olanzapine to other atypical antipsychotics and track the outcomes.
